# Supplementary material for: Unveiling the Bioactive Potential of the Invasive Jellyfish Phyllorhiza punctata Through Integrative Transcriptomic and Proteomic Analyses
Source: Biomolecules. 2025 Aug 4;15(8):1121. doi: 10.3390/biom15081121 (PMC12383608; doi:10.3390/biom15081121)
Supplement: Supplementary file 1 [file biomolecules-15-01121-s001.zip › Suplementary Material/FileS3/PEPFOLD4/XP_065052795_7208/clusters.html]

 PEP-FOLD clusters


Download archive of all models  here   

|  |  |  |
| --- | --- | --- |
| # | file | sOPEP |
| # cluster 1 - Size: 33 |  |  |
|  | XP\_065052795\_7208-model1 | -11.3291 |
|  | XP\_065052795\_7208-model1.1 | -11.3291 |
|  | XP\_065052795\_7208-model1.2 | -11.3291 |
|  | XP\_065052795\_7208-model1.3 | -11.3291 |
|  | XP\_065052795\_7208-model1.4 | -11.3191 |
|  | XP\_065052795\_7208-model1.5 | -11.2394 |
|  | XP\_065052795\_7208-model1.6 | -11.2394 |
|  | XP\_065052795\_7208-model1.7 | -11.2394 |
|  | XP\_065052795\_7208-model1.8 | -11.2394 |
|  | XP\_065052795\_7208-model1.9 | -11.2394 |
|  | XP\_065052795\_7208-model1.10 | -11.2394 |
|  | XP\_065052795\_7208-model1.11 | -11.2394 |
|  | XP\_065052795\_7208-model1.12 | -11.2394 |
|  | XP\_065052795\_7208-model1.13 | -11.2394 |
|  | XP\_065052795\_7208-model1.14 | -11.2394 |
|  | XP\_065052795\_7208-model1.15 | -11.2394 |
|  | XP\_065052795\_7208-model1.16 | -11.2394 |
|  | XP\_065052795\_7208-model1.17 | -11.2394 |
|  | XP\_065052795\_7208-model1.18 | -11.2394 |
|  | XP\_065052795\_7208-model1.19 | -11.2394 |
|  | XP\_065052795\_7208-model1.20 | -11.2394 |
|  | XP\_065052795\_7208-model1.21 | -11.2394 |
|  | XP\_065052795\_7208-model1.22 | -11.2394 |
|  | XP\_065052795\_7208-model1.23 | -11.2394 |
|  | XP\_065052795\_7208-model1.24 | -11.2394 |
|  | XP\_065052795\_7208-model1.25 | -11.2394 |
|  | XP\_065052795\_7208-model1.26 | -11.2387 |
|  | XP\_065052795\_7208-model1.27 | -11.2387 |
|  | XP\_065052795\_7208-model1.28 | -11.2387 |
|  | XP\_065052795\_7208-model1.29 | -11.1842 |
|  | XP\_065052795\_7208-model1.30 | -11.1821 |
|  | XP\_065052795\_7208-model1.31 | -11.176 |
|  | XP\_065052795\_7208-model1.32 | -11.1476 |
| # cluster 2 - Size: 9 |  |  |
|  | XP\_065052795\_7208-model2 | -4.44081 |
|  | XP\_065052795\_7208-model2.1 | -4.41577 |
|  | XP\_065052795\_7208-model2.2 | -4.37954 |
|  | XP\_065052795\_7208-model2.3 | -4.36447 |
|  | XP\_065052795\_7208-model2.4 | -4.21055 |
|  | XP\_065052795\_7208-model2.5 | -3.62284 |
|  | XP\_065052795\_7208-model2.6 | -3.35822 |
|  | XP\_065052795\_7208-model2.7 | -3.27155 |
|  | XP\_065052795\_7208-model2.8 | -2.95852 |
| # cluster 3 - Size: 30 |  |  |
|  | XP\_065052795\_7208-model3 | -3.23589 |
|  | XP\_065052795\_7208-model3.1 | -3.16611 |
|  | XP\_065052795\_7208-model3.2 | -3.15518 |
|  | XP\_065052795\_7208-model3.3 | -3.13113 |
|  | XP\_065052795\_7208-model3.4 | -3.10961 |
|  | XP\_065052795\_7208-model3.5 | -3.08849 |
|  | XP\_065052795\_7208-model3.6 | -3.05004 |
|  | XP\_065052795\_7208-model3.7 | -3.05004 |
|  | XP\_065052795\_7208-model3.8 | -3.03314 |
|  | XP\_065052795\_7208-model3.9 | -3.03007 |
|  | XP\_065052795\_7208-model3.10 | -3.02011 |
|  | XP\_065052795\_7208-model3.11 | -3.0075 |
|  | XP\_065052795\_7208-model3.12 | -2.99373 |
|  | XP\_065052795\_7208-model3.13 | -2.97339 |
|  | XP\_065052795\_7208-model3.14 | -2.9376 |
|  | XP\_065052795\_7208-model3.15 | -2.93629 |
|  | XP\_065052795\_7208-model3.16 | -2.92264 |
|  | XP\_065052795\_7208-model3.17 | -2.91023 |
|  | XP\_065052795\_7208-model3.18 | -2.90478 |
|  | XP\_065052795\_7208-model3.19 | -2.89821 |
|  | XP\_065052795\_7208-model3.20 | -2.88442 |
|  | XP\_065052795\_7208-model3.21 | -2.859 |
|  | XP\_065052795\_7208-model3.22 | -2.85234 |
|  | XP\_065052795\_7208-model3.23 | -2.83755 |
|  | XP\_065052795\_7208-model3.24 | -2.83489 |
|  | XP\_065052795\_7208-model3.25 | -2.79815 |
|  | XP\_065052795\_7208-model3.26 | -2.60609 |
|  | XP\_065052795\_7208-model3.27 | -2.5886 |
|  | XP\_065052795\_7208-model3.28 | -2.52516 |
|  | XP\_065052795\_7208-model3.29 | -2.3915 |
| # cluster 4 - Size: 14 |  |  |
|  | XP\_065052795\_7208-model4 | -3.1138 |
|  | XP\_065052795\_7208-model4.1 | -3.11004 |
|  | XP\_065052795\_7208-model4.2 | -3.10229 |
|  | XP\_065052795\_7208-model4.3 | -3.08811 |
|  | XP\_065052795\_7208-model4.4 | -3.00586 |
|  | XP\_065052795\_7208-model4.5 | -3.00586 |
|  | XP\_065052795\_7208-model4.6 | -2.99117 |
|  | XP\_065052795\_7208-model4.7 | -2.98146 |
|  | XP\_065052795\_7208-model4.8 | -2.90125 |
|  | XP\_065052795\_7208-model4.9 | -2.83537 |
|  | XP\_065052795\_7208-model4.10 | -2.77989 |
|  | XP\_065052795\_7208-model4.11 | -2.70708 |
|  | XP\_065052795\_7208-model4.12 | -2.65654 |
|  | XP\_065052795\_7208-model4.13 | -2.57318 |
| # cluster 5 - Size: 1 |  |  |
|  | XP\_065052795\_7208-model5 | -2.94772 |
| # cluster 6 - Size: 1 |  |  |
|  | XP\_065052795\_7208-model6 | -2.1815 |
| # cluster 7 - Size: 2 |  |  |
|  | XP\_065052795\_7208-model7 | -1.97356 |
|  | XP\_065052795\_7208-model7.1 | -1.85929 |
| # cluster 8 - Size: 2 |  |  |
|  | XP\_065052795\_7208-model8 | -1.9532 |
|  | XP\_065052795\_7208-model8.1 | -1.90222 |
| # cluster 9 - Size: 2 |  |  |
|  | XP\_065052795\_7208-model9 | -1.94022 |
|  | XP\_065052795\_7208-model9.1 | -1.88842 |
| # cluster 10 - Size: 1 |  |  |
|  | XP\_065052795\_7208-model10 | -1.93319 |
| # cluster 11 - Size: 2 |  |  |
|  | XP\_065052795\_7208-model11 | -1.90791 |
|  | XP\_065052795\_7208-model11.1 | -1.80138 |
| # cluster 12 - Size: 2 |  |  |
|  | XP\_065052795\_7208-model12 | -1.88836 |
|  | XP\_065052795\_7208-model12.1 | -1.86535 |
| # cluster 13 - Size: 1 |  |  |
|  | XP\_065052795\_7208-model13 | -1.8737 |
|  |  |  |
